# Geriatric 8 score predicts surgical risk and oncologic outcomes in older patients with upper tract urothelial carcinoma undergoing radical nephroureterectomy

**DOI:** 10.1186/s12877-026-07954-1

**Published:** 2026-07-07

**Authors:** Chien-Hsiung Lo, Cheng-Han Chang, Che-Yuan Hu, Yin-Chien Ou, Kun-Che Lin, Yuh-Shyan Tsai, Hau-Chern Jan, Kuan-Yu Wu

**Affiliations:** 1 Department of Urology, National Cheng Kung University Hospital, College of Medicine, National Cheng Kung University, No.138, Sheng-Li Road, Tainan, 70403 Taiwan, R. O. C.; 2 Department of Medicine, College of Medicine, National Cheng Kung University, Tainan, Taiwan; 3 Division of Urology, Department of Surgery, National Cheng Kung University Hospital Dou-Liou Branch, Yunlin, Taiwan

**Keywords:** Frailty, Geriatric 8, Prognosis, Postoperative complications, Radical nephroureterectomy, Upper tract urothelial carcinoma

## Abstract

**Background:**

Accurate preoperative risk stratification remains challenging in older patients with upper tract urothelial carcinoma (UTUC) undergoing radical nephroureterectomy (RNU). This study evaluated whether preoperative Geriatric 8 (G8) score is associated with perioperative and oncologic outcomes.

**Methods:**

We retrospectively analyzed 338 patients aged ≥ 65 years who underwent RNU between 2013 and 2022. Patients were classified as G8-low (score ≤ 14) or G8-high (score > 14). Multivariable logistic and Cox regression analyses assessed the association between frailty, postoperative complications (Clavien–Dindo classification), and survival outcomes, including overall survival (OS), cancer-specific survival (CSS), and progression-free survival (PFS).

**Results:**

Overall, 178 patients (52.7%) were G8-low. G8-low patients exhibited significantly longer hospital stays and higher rates of minor (Clavien I-II: 60.1% vs. 35.6%, *p* < 0.001) and major complications (Clavien III-V: 12.4% vs. 3.7%, *p* = 0.004). Multivariable analysis identified G8-low as an independent predictor of postoperative complications (OR 2.51; 95% CI 1.50–4.20; *p* < 0.001), demonstrating superior prognostic value compared to ASA classification. Furthermore, G8-low remained an independent predictor of worse OS (HR 1.76, 95% CI 1.17–2.65; *p* = 0.006), CSS (HR 2.00, 95% CI 1.31–3.07; *p* = 0.001), and PFS (HR 1.73, 95% CI 1.20–2.50; *p* = 0.004).

**Conclusions:**

Preoperative G8-low status, is independently associated with increased postoperative complications and poorer oncologic outcomes in older UTUC patients undergoing RNU. Incorporating G8 screening into routine preoperative evaluation may facilitate accurate risk stratification, aiding shared decision-making regarding radical versus nephron-sparing approaches.

**Supplementary Information:**

The online version contains supplementary material available at https://doi.org/10.1186/s12877-026-07954-1.

## Background

Urothelial carcinoma (UC) originates from the urothelial lining of the renal pelvis, ureter, bladder, and urethra. While upper tract urothelial carcinoma (UTUC)—involving the renal pelvis and ureter—accounts for only 5–10% of all UC cases globally [1], its epidemiology in Taiwan is unique. The incidence and prevalence in Taiwan are remarkably high, accounting for 20–30% of all UC diagnoses [2]. This distinct epidemiological profile is strongly linked to environmental exposures, specifically arsenic in artesian well water and aristolochic acid, alongside a high prevalence of end-stage renal disease (ESRD) [3–5]. Compared with bladder cancer, UTUC is typically diagnosed at a more advanced stage and is associated with poorer oncologic outcomes [6].

With the progressive aging of populations worldwide, the age at UTUC diagnosis has steadily increased. Chen et al. reported an increase in mean age from 70.0 years in 1988 to 73.2 years in 2015 [7], while U.S. data showed a similar increase from 68 to 73 years over the past three decades [8]. Data from the Taiwan Cancer Registry confirm a similar rise in age-specific UTUC incidence, underscoring the growing clinical burden of managing older patients with UTUC [9].

Radical nephroureterectomy (RNU) remains the standard treatment for localized UTUC. However, it is a major procedure associated with substantial perioperative morbidity, particularly in older patients with limited physiological reserve. Despite advances in surgical techniques, complication rates remain approximately 10–12%, highlighting the limitations of conventional clinicopathologic parameters for preoperative risk stratification [10]. In this demographic, chronological age alone is often an unreliable predictor of surgical tolerance. Consequently, “frailty”—a clinical state of reduced physiological reserve and heightened vulnerability to stressors—has emerged as a critical prognostic factor for postoperative complications and mortality in geriatric oncology [11–14]. Identifying frail patients is therefore essential to balance oncologic benefits against surgical risks and to facilitate shared decision-making [15].

The comprehensive geriatric assessment (CGA) is considered the gold standard for diagnosing frailty, evaluating physical, cognitive, and functional domains. However, its complexity and time-intensive nature limit its routine application in busy surgical practices [11, 16]. To address this, the Geriatric 8 (G8) screening tool was developed as a rapid, practical alternative. The G8 is an eight-item questionnaire assessing age, body mass index (BMI), nutritional status, mobility, neuropsychological status, polypharmacy, and self-perceived health. With a score range of 0–17, a cut-off of ≤ 14 has demonstrated high sensitivity (85%) for detecting frailty compared to the CGA [17–20].

While the G8 has been validated as a prognostic tool in various malignancies—including gastrointestinal, lung, and prostate cancers [21–23], as well as in predicting postoperative complications for major uro-oncologic surgeries [24–26] its application in UTUC remains largely unexplored. Previous studies in UTUC have primarily utilized simplified metrics, such as the 5-item frailty index, which focuses mainly on comorbidities and functional dependence [27]. In contrast, the G8 encompasses broader geriatric domains, including nutritional and neuropsychological status, potentially offering a more comprehensive preoperative risk stratification. Therefore, this study aims to evaluate the G8 score as a predictor of surgical risk and oncologic outcomes in older patients with UTUC undergoing RNU, providing evidence to optimize perioperative management in this specific population.

## Patients and methods

### Study population

We retrospectively identified patients aged ≥ 65 years who underwent RNU for UTUC in Taiwan between January 2013 and December 2022. Eligible patients met the following criteria: (1) age ≥ 65 years at surgery; (2) receipt of RNU; (3) histopathologically confirmed urothelial carcinoma; (4) no evidence of distant metastasis at diagnosis; and (5) availability of complete clinical and follow-up data. Patients with incomplete medical records were excluded.

### Data collection and definition of variables

Baseline demographics, clinicopathological and perioperative data were collected, including age, sex, comorbidities, surgical approach, tumor stage, histological grade, lymphovascular invasion, adjuvant chemotherapy, estimated blood loss, operative time, length of hospital stay, postoperative complications, and 30-day readmission rates. Postoperative complications were classified according to the Clavien–Dindo classification, with Grades I–II defined as minor and Grades III–V as major complications. It should be noted that due to our institutional documentation protocol, which primarily captures complications requiring pharmacological, surgical, or radiological interventions, Grade I events (deviations from the normal course without need for treatment) were not systematically recorded in this cohort.

Oncologic outcomes included progression-free survival (PFS), cancer-specific survival (CSS), and overall survival (OS). Preoperative vulnerability was assessed using the Geriatric 8 (G8) screening tool. In our institution, all patients scheduled for radical nephroureterectomy are routinely admitted 1–2 days before surgery and undergo a standardized nursing admission assessment. This routine point-of-care assessment prospectively captures all eight G8 domains: recent weight loss, appetite changes, BMI, mobility, neuropsychological status, polypharmacy (> 3 medications), self-perceived health compared with peers, and age. Notably, subjective items are elicited directly from the patients by admitting nurses at the bedside and recorded in structured fields within the electronic medical record. Therefore, in the present study, G8 data were retrospectively abstracted from these prospectively and routinely documented records, rather than reconstructed from incomplete chart information. To minimize observer bias, the data abstraction was performed using a standardized template by an investigator blinded to the patients’ postoperative outcomes. Patients with missing data in any of the eight domains were excluded from the final analysis to ensure the accuracy of the G8 scoring. Total G8 scores range from 0 to 17, with lower scores indicating greater vulnerability. In accordance with the original validation studies, patients with a G8 score of ≤ 14 were classified as G8-low (screen-positive for vulnerability), whereas those with a G8 score of > 14 were classified as G8-high.

### Statistical analysis

All statistical analyses were performed using SPSS (v29.0.2.0; IBM Corp., Chicago, IL, USA). Continuous variables were compared with independent t-tests, and categorical variables with chi-square tests. Univariable and multivariable logistic regression analyses were conducted to identify predictors of postoperative complications. Survival (OS, CSS, PFS) was estimated with Kaplan–Meier method and compared with log-rank test. Cox proportional hazards regression models were applied to evaluate prognostic factors associated with survival outcomes. Candidate variables were first examined in univariable analyses, and variables with *p* < 0.15 and/or considered clinically relevant were entered into the multivariable models. To prevent model overfitting, the number of included covariates was restricted according to the widely accepted rule of approximately 10 outcome events per variable (EPV) [28, 29], ensuring that the final models were adequately supported by the available events. All statistical tests were two-sided, and p values < 0.05 were considered statistically significant.

## Results

### Baseline clinical characteristics

A total of 338 patients with UTUC who underwent RNU were analyzed. The mean age was 74.3 ± 7.7 years, and the median follow-up duration was 36.8 months (IQR, 40.7 months). 178 patients (52.7%) were G8-low, and 160 (47.3%) were G8-high. Clinicopathological characteristics and unadjusted group comparisons are summarized in Table 1.

**Table 1:** Baseline characteristics of the cohort

Variable	All	G8-high (score > 14)	G8-low (score ≤ 14)	*p*-value
	*N* = 338	*N* = 160 (47.3%)	*N* = 178 (52.7%)	
Age at diagnosis (yr), mean ± SD	74.3 ± 7.7	72.5 ± 7.0	76.0 ± 7.9	**< 0.001**
Follow-up time after RNU (mo), median (IQR)	36.8 (40.7)	50.1 (32.6)	37.6 (29.2)	**< 0.001**
Sex, N(%)				**0.04**
Female	199 (58.9%)	85 (53.1%)	114 (64.0%)	
Male	139 (41.1%)	75 (46.9%)	64 (36.0%)	
Renal function (ml/min/1.73m^2^), N(%)				**0.008**
eGFR ≥ 45	178 (52.7%)	96 (60.0%)	82 (46.1%)	
eGFR < 45	104 (30.8%)	47 (29.4%)	57 (32.0%)	
ESRD	56 (16.5%)	17 (9.6%)	39 (21.9%)	
Hypertension, N(%)	215 (63.6%)	102 (63.7%)	113 (63.5%)	0.96
Diabetes Mellitus, N(%)	114 (33.7%)	41 (23.6%)	73 (41.0%)	**0.003**
Previous BC or Concomitant BC, N(%)	104 (30.8%)	47 (29.4%)	57 (32.0%)	0.60
Surgical approach, N(%)				0.86
Open	34 (11.2%)	17 (10.6%)	17(9.6%)	
Minimally-invasive	304 (89.8%)	143 (89.4%)	161 (90.4%)	
ASA ≥ 3, N(%)	204 (60.4%)	76 (47.5%)	128 (71.9%)	**< 0.001**
Tumor size ≥ 2 cm N(%)	247 (73.1%)	112 (70.0%)	135 (75.8%)	0.23
Tumor location, N(%)				0.45
Pelvis	149 (44.1%)	70 (43.8%)	79 (44.4%)	
Ureter	128 (37.9%)	65 (40.6%)	63 (35.4%)	
Both	61 (18.0%)	25 (15.6%)	36 (20.2%)	
Pathologic T stage, N(%)				0.58
Tis/a/1	123 (36.4%)	59 (36.9%)	64 (36.0%)	
T2	63 (18.6%)	33 (20.6%)	30 (16.9%)	
T3/4	152 (45.0%)	68 (42.5%)	84 (47.2%)	
Lymph node status, N(%)				0.83
N0/x	322 (95.3%)	152 (95.0%)	170 (95.6%)	
N+	16 (4.7%)	8 (5.0%)	8 (4.4%)	
Tumor grade, N(%)				0.45
Low	14 (4.1%)	8 (5.0%)	6 (3.4%)	
High	324 (95.9%)	152 (95.0%)	172 (96.6%)	
Lymphovascular invasion, N(%)	96 (28.4%)	43 (22.9%)	53 (29.8%)	0.56
Multifocality, N(%)	83 (24.6%)	35 (21.9%)	48 (27.0%)	0.28
Adjuvant chemotherapy	62 (18.5%)	32 (20.0%)	30 (16.9%)	0.57

*Abbreviations* : *RNU *radical nephroureterectomy, *BC *bladder cancer, *ASA *American Society of Anesthesiologists, *eGFR *estimated glomerular filtration rate, *ESRD *end-stage renal disease, *SD *standard deviation, *IQR *interquartile range

% is expressed as the percentage of G8-high or G8-low group

Bold *p* values indicate statistical significance at *p* < 0.05

G8-low patients were significantly older (76.0 ± 7.9 vs. 72.5 ± 7.0 years, *p* < 0.001), more frequently female (64.0% vs. 53.1%, *p* = 0.042), and exhibited a higher prevalence of DM (41.0% vs. 23.6%, *p* = 0.003), worse renal function (*p* = 0.008), and a higher proportion of American Society of Anesthesiologists (ASA) class ≥ 3 (*p* < 0.001) when compared to G8-high patients. In contrast, tumor-related characteristics—including pathological T stage, size, location, nodal status, grade, lymphovascular invasion, and multifocality—did not differ significantly between the two groups.

### Comparison of perioperative outcomes between the G8-low and G8-high groups

Perioperative outcomes are summarized in Table 2. A total of 173 patients experienced at least one postoperative complication within 30 days. At the patient level, 164 (48.5%) experienced at least one grade II complication, 20 (5.9%) experienced at least one grade III complication, and 14 (4.2%) experienced at least one grade IV complication. No grade V complications were captured. Detailed event-level categories are provided in Table S1. Operative time and estimated blood loss did not differ between G8-low and G8-high patients. However, G8-low patients experienced significantly longer hospital stays (11.2 ± 6.2 vs. 8.7 ± 2.3 days, *p* < 0.001) and higher complication rates. Specifically, G8-low patients had increased rates of minor complications (60.1% vs. 35.6%, *p* < 0.001), major complications (12.4% vs. 3.7%, *p* = 0.004), grade III complications (9.0% vs. 2.5%, *p* = 0.011), and grade IV complications (6.8% vs. 1.3%, *p* = 0.011). In addition, blood transfusion was more frequently required in G8-low patients (46.0% vs. 22.0%, *p* < 0.001). Rates of postoperative fever and 30-day readmission did not differ significantly between groups.

**Table 2:** Comparison of Peri-operative outcome and complications between the G8-low and G8-high groups

	All	G8-high (score>14)	G8-low (score≤14)	P-value
	N=338	N=160	N=178	
Operative time (min), mean ± SD	225 ± 134	224 ± 132	226 ± 137	0.85
Blood loss (mL), mean ± SD	285 ± 674	243 ± 379	317 ± 846	0.31
Length of stay (days), mean ± SD	10.0 ± 5.0	8.7 ± 2.3	11.2 ± 6.2	**<0.001**
Minor complications^1,2^N(%)	164 (48.5%)	57 (35.6%)	107 (60.1%)	**<0.001**
Major complications^1,2^,N(%)	28 (8.3%)	6 (3.7%)	22 (12.4%)	**0.004**
Grade 3 complications^2^,N(%)	20 (5.9%)	4 (2.5%)	16 (9.0%)	**0.01**
Grade 4 complications^2^,N(%)	14 (4.2%)	2 (1.3%)	12 (6.8%)	**0.01**
Transfusion, N(%)	116 (34.6%)	35 (22.0%)	81 (46.0%)	**<0.001**
Fever, N(%)	46 (13.7%)	20 (12.6%)	26 (14.7%)	0.57
30-day readmission, N(%)	13 (3.8%)	4 (2.5%)	9 (5.1%)	0.22

1 Major complications defined as Clavien‒Dindo classification grade ≥3; while minor complications as Clavien‒Dindo classification grade 1 or 2

2 Represent the proportion of patients experiencing at least one complication of each grade category

*Abbreviation*: *SD *standard deviation

% is expressed as the percentage of G8-high or G8-low group

Bold *p* values indicate statistical significance at *p* < 0.05

## Predictors of postoperative complications

Logistic regression analysis identified independent predictors of postoperative complications (Table 3). In univariate analysis, age ≥ 80 years, greater intraoperative blood loss, prolonged operative time (≥ 240 min), higher ASA class (≥ 3), end-stage renal disease (ESRD), DM, and G8-low were all significantly associated with an increased risk of postoperative complications (all *p* < 0.05).

**Table 3:** Univariate and multivariate logistic regression analyses identifying risk factors for postoperative complications

Variables		Univariate		Multivariate	
		OR (95% CI)	*p*-value	OR (95% CI)	*p*-value
Age (≥ 80 vs. <80)		2.08 (1.35–3.20)	< 0.001	2.03 (1.22–3.37)	0.006
Blood loss		1.002 (1.001–1.003)	< 0.001	1.002 (1.001–1.004)	< 0.001
Operative time (min) (≥ 240 vs. <240)		1.75 (1.13–2.71)	0.01	1.61 (0.91–2.83)	0.10
ASA (≥ 3 vs. <3)		1.66 (1.07–2.58)	0.02	1.05 (0.61–1.80)	0.86
Gender (Male vs. Female)		0.77 (0.50–1.18)	0.23	-	
Renal function (ml/min/1.73m2)					
eGFR ≥ 45		Reference		Reference	
eGFR < 45		1.52 (0.93–2.47)	0.09	1.48 (0.84–2.60)	0.18
ESRD		2.53 (1.36–4.72)	0.003	3.20 (1.49–6.89)	0.003
HTN (Yes vs. No)		0.94 (0.60–1.46)	0.77	-	
DM (Yes vs. No)		2.20 (1.39–3.49)	< 0.001	1.81 (1.04–3.15)	0.04
Surgical Approach (Open vs. MIS)		1.07 (0.53–2.17)	0.86	-	
Pathological T stage					
G8-status (low vs. high)		2.72 (1.75–4.23)	< 0.001	2.51 (1.50–4.20)	< 0.001

*Abbreviations*: *ASA *American Society of Anesthesiologists, *eGFR *estimated glomerular filtration rate, *ESRD *end-stage renal disease, *HTN *hypertension, *DM *diabetes mellitus, *MIS *minimally-invasive surgery, *OR *odds ratio, *CI *confidence interval

In the multivariable model, independent predictors included age ≥ 80 years (OR 2.028, 95% CI 1.221–3.367; *p* = 0.006), intraoperative blood loss (OR 1.002 per mL, 95% CI 1.001–1.004; *p* < 0.001), ESRD (OR 3.201, 95% CI 1.488–6.886; *p* = 0.003), DM (OR 1.807, 95% CI 1.036–3.152; *p* = 0.037), and G8-low (OR 2.514, 95% CI 1.503–4.203; *p* < 0.001). Conversely, ASA classification did not reach statistical significance in the multivariable analysis. These findings highlight G8-low, renal failure, blood loss, advanced age, and DM as key determinants of short-term postoperative morbidity following RNU.

## G8 score as an independent predictor of oncologic outcomes

**Fig. 1:**
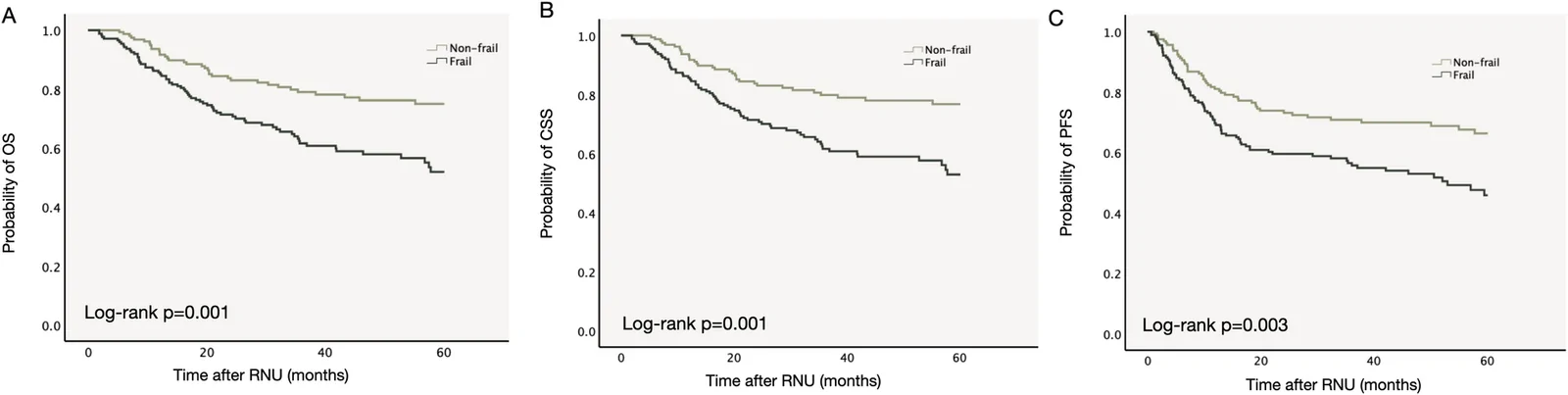
Kaplan–Meier estimates of survival outcomes in patients undergoing radical nephroureterectomy, stratified by G8 score. The gray line represents the G8-high (score > 14) group, and the black line represents the G8-low (score ≤ 14) group. **A** Overall survival (OS). **B** Cancer-specific survival (CSS). **C** Progression-free survival (PFS)

During the follow-up period, a total of 115 overall deaths, 108 cancer-specific deaths, and 138 disease progression events were observed. Kaplan–Meier survival analysis demonstrated that G8-low patients had significantly worse OS (*p* = 0.001), CSS (*p* = 0.001), and PFS (*p* = 0.003) than G8-high patients (Figure 1).

**Fig. 2:**
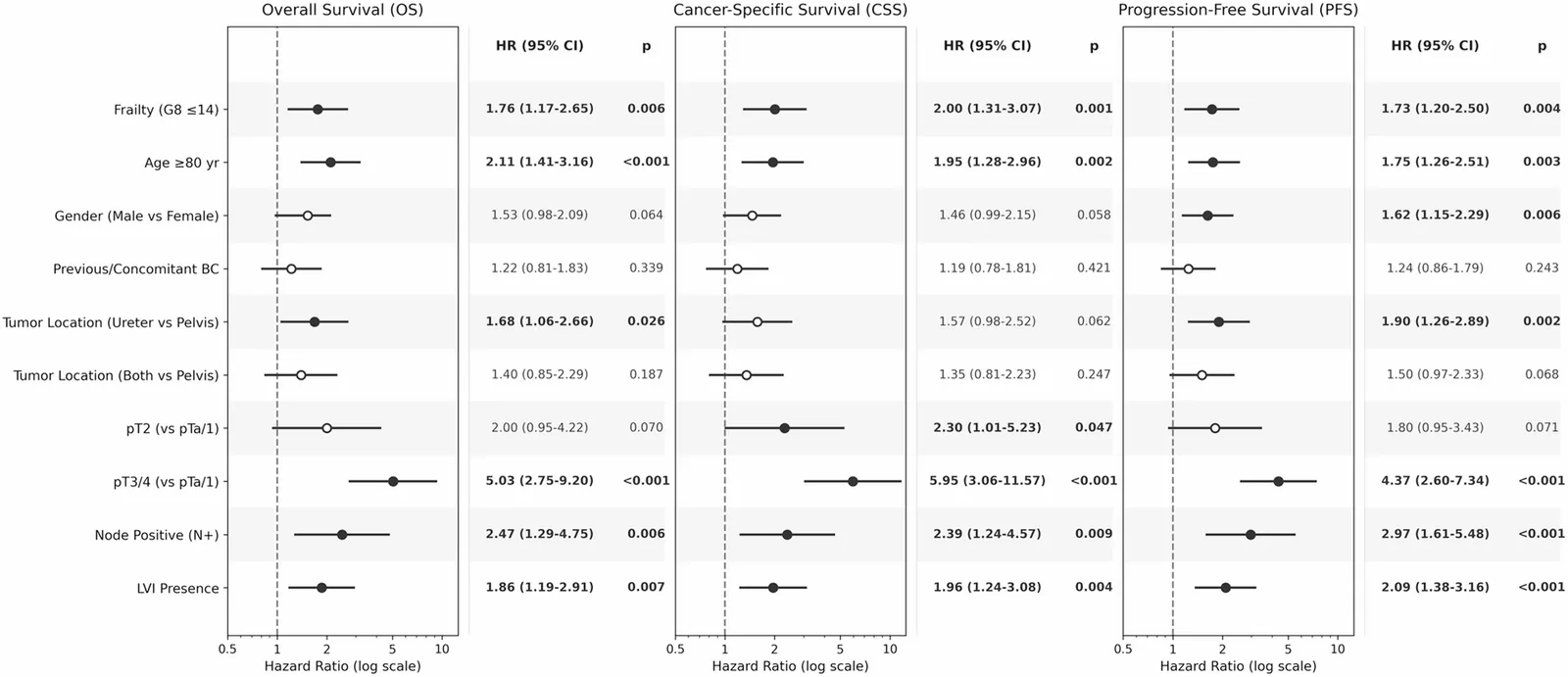
Forest plot of multivariable Cox proportional hazards regression analyses predicting oncologic outcomes. The analysis evaluates Overall Survival (OS), Cancer-Specific Survival (CSS), and Progression-Free Survival (PFS). The model is adjusted for G8-status, age, gender, previous/concomitant bladder cancer (BC), tumor location, pathological T stage (pT), nodal status, lymphovascular invasion (LVI), and adjuvant chemotherapy. Filled markers and bold text indicate statistical significance (*p* < 0.05). Data are presented as adjusted hazard ratios (HR) with 95% confidence intervals (CI) on a log scale. The dashed vertical line represents the reference (HR = 1.0). Abbreviations: BC = bladder cancer; LVI = lymphovascular invasion; pT = pathological T stage

Cox proportional hazards regression analyses were performed to identify prognostic factors for oncologic outcomes, with results summarized in Fig. 2 and detailed in additional files 2–4: Tables S2–S4. On univariable analysis, age ≥ 80 year, male sex, advanced pathological T stage, nodal involvement, lymphovascular invasion, adjuvant chemotherapy, and G8-low were significantly associated with inferior OS, CSS, and PFS. In multivariable models, age ≥ 80 year, higher pathological T stage, node-positive disease, and lymphovascular invasion remained independent predictors of worse OS, CSS, and PFS. Notably, G8-low was confirmed as an independent predictor of worse OS (HR 1.77, 95% CI 1.18–2.67; *p* = 0.006), CSS (HR 2.02, 95% CI 1.32–3.10; *p* = 0.001), and PFS (HR 1.70, 95% CI 1.18–2.46; *p* = 0.005). In addition, ureteral tumor location independently predicted poorer OS and PFS, while male sex and adjuvant chemotherapy were independently associated with inferior PFS.

## Discussion

The present study demonstrates the prognostic value of the G8 screening tool for both surgical risk stratification and oncologic outcomes in older UTUC patients undergoing RNU. In our cohort, with a mean age of 74.3 ± 7.7 years, more than half (52.7%) were classified as G8-low. G8-low was significantly associated with increased postoperative complications, prolonged hospital stays, higher transfusion rates, and inferior survival outcomes, including OS, CSS, and PFS. These findings underscore the clinical necessity of incorporating geriatric assessment into routine preoperative evaluation for this vulnerable population.

G8-low patients experienced significantly higher rates of both minor and major postoperative complications, reflecting diminished physiological reserve and heightened vulnerability to surgical stress. Among minor complications, the most pronounced differences were observed in blood transfusion requirements (45.5% vs. 21.9%), followed by postoperative delirium (6.2% vs. 1.3%). For major complications, the incidence of postoperative shock was notably higher in the G8-low group (4.5% vs. 0.6%; Table S1). Importantly, G8-low remained an independent predictor of complications after adjustment for confounders including advanced age, ESRD, DM, and intraoperative blood loss. This confirms the robustness of the G8 score as a perioperative risk stratification tool.

The association between G8-low and postoperative delirium deserves specific attention. The heightened burden of postoperative delirium among frail patients is well documented [30, 31]. Frailty is postulated to increase delirium risk through mechanisms involving chronic systemic inflammation and compromised physiological and cognitive reserves, thereby diminishing resilience to surgery-induced neuroinflammatory stress [32, 33]. Consistent with prior work, our study observed a higher incidence of postoperative delirium among G8-low patients. These results underscore the need for routine frailty assessment and targeted perioperative strategies—such as delirium screening, optimization of comorbidities, and early involvement of multidisciplinary geriatric care—to mitigate delirium risk in this vulnerable population.

Noteworthy is the comparison between the G8 score and the ASA classification. While the ASA classification is a well-established predictor of surgical risk [34, 35], our multivariate analysis demonstrated that its predictive value was superseded by the inclusion of G8 status. A similar pattern was observed in a prior study, where ASA was significant only in univariate but not multivariate analysis [36]. This suggests that the G8 score serves as a superior surrogate for physiological reserve in geriatric UTUC patients, as it captures critical vulnerabilities like malnutrition and functional decline that the ASA score may overlook.

Beyond postoperative morbidity, the G8 score also demonstrated significant prognostic value for oncologic outcomes. Multivariate Cox regression analysis demonstrated that G8-low independently predicted OS, CSS, and PFS, even after adjustment for established prognostic factors such as tumor stage, nodal involvement, and lymphovascular invasion. These findings align with accumulating evidence across multiple malignancies, indicating that frailty is an independent determinant of cancer prognosis [19]. The mechanism is likely multifactorial: reduced physiological reserve increases susceptibility to surgical stress and postoperative complications, including bleeding, infection, and metabolic disturbances. Nutritional impairment—manifested by weight loss, low body mass index, and poor appetite—compromises immune function and wound healing, while chronic inflammation and polypharmacy further hinder postoperative recovery and tolerance to adjuvant therapies. Collectively, these factors contribute not only to increased surgical morbidity but also to inferior long-term survival, potentially reflecting impaired host immunity or more aggressive tumor biology [37].

Notably, the proportion of patients receiving adjuvant systemic therapy in our cohort was relatively low, with only 62 of 338 patients (18.5%) undergoing postoperative treatment. This pattern can be explained by two main factors. First, in routine clinical practice, adjuvant systemic therapy is primarily indicated for patients harboring high-risk, locally advanced disease (i.e., pathological stage ≥ pT3 and/or node-positive disease). Second, this utilization pattern should be interpreted in light of the study period, which extended from January 2013 to December 2022. The phase 3 POUT trial, which provided pivotal randomized evidence supporting adjuvant platinum-based chemotherapy in UTUC, and the CheckMate 274 trial, which established the role of adjuvant nivolumab, were not published until April 2020 and June 2021, respectively [38, 39]. Consequently, guideline recommendations were significantly strengthened only toward the end of our study period. In our study, all 62 patients who received adjuvant therapy underwent chemotherapy; this is because Taiwan’s National Health Insurance only reimburses adjuvant therapy for patients with stage pT3 disease or higher, whereas the use of nivolumab requires out-of-pocket payment.

Furthermore, in our multivariable Cox regression analysis, adjuvant therapy was associated with worse PFS rather than a protective effect. This finding most likely reflects confounding by indication, since patients who received adjuvant therapy were preferentially those with adverse pathological features, including advanced T stage, nodal disease, and lymphovascular invasion, all of which are intrinsically associated with inferior oncologic outcomes. Most importantly, even after adjustment for the receipt of adjuvant therapy, G8-low status remained a robust, independent predictor of worse OS, CSS, and PFS.

The prognostic capability of frailty indices is established in UTUC. Previous studies using simplified frailty indices, such as the five-item frailty index (5-item FI), have reported associations with postoperative complications and survival in UTUC patients [27]. Similarly, frailty assessed by the Johns Hopkins frailty indicator independently predicted overall complications, in-hospital mortality, non-home-based discharge, and prolonged hospitalization [40]. Nutritional indices such as the Geriatric Nutritional Risk Index (GNRI) have also been identified as significant prognostic indicators in UTUC, further emphasizing the importance of incorporating geriatric parameters in oncologic risk stratification [41]. However, these indices predominantly capture isolated domains such as comorbidity burden, functional dependence, or nutritional status. In contrast, the G8 provides a multidimensional assessment encompassing nutritional status, polypharmacy, psychological health, and self-perceived well-being, thus delivering a more comprehensive evaluation of frailty. This broader scope likely explains the strong prognostic performance in our study.

From a clinical perspective, our findings support incorporating the G8 into routine preoperative evaluation for older UTUC patients. As a simple and time-efficient screening tool, the G8 overcomes the practical limitations of comprehensive geriatric assessment while maintaining substantial predictive value, thereby facilitating the incorporation of G8-based risk stratification into surgical decision-making. Early identification of potentially frail patients allows for tailored interventions—such as nutritional optimization, physical prehabilitation, and management of comorbidities—to attenuate perioperative morbidity. Furthermore, frailty screening may shape postoperative management strategies, including intensified surveillance, early geriatric co-management, and individualized adjuvant therapies, while aiding the selection of candidates for less invasive modalities like Nephron-Sparing Surgery (NSS).

According to the European Association of Urology guidelines, NSS—including segmental ureterectomy and endoscopic management—should be considered the treatment option for low-risk UTUC [42]. A large systematic review demonstrated that, compared with RNU, NSS confers superior preservation of renal function. While segmental ureterectomy offers comparable oncologic outcomes, endoscopic management is associated with a higher rate of local recurrence [43]. Although direct comparative data remain limited, a meta-analysis reported a pooled complication rate of 12.5% for retrograde endoscopic management [44], whereas RNU carries an overall complication rate ranging from 11.9% to 43.8% [45]. Given the limited evidence regarding complications specifically in frail patients, shared decision-making is paramount to balance surgical benefits against potential risks. Moreover, the emergence of novel systemic therapies (e.g., antibody-drug conjugates and immunotherapy) may further expand the indication for NSS within multimodal neoadjuvant or adjuvant treatment paradigms.

Several limitations of this study should be acknowledged. First, the retrospective, single-center design limits generalizability and introduces selection bias by potentially excluding severely frail patients less likely to undergo surgery. In addition, the unique epidemiology of UTUC in Taiwan—characterized by a high prevalence of aristolochic acid exposure and ESRD—may restrict external validity. Second, although a G8 cutoff of ≤ 14 has been validated across multiple cancer populations, the optimal threshold for UTUC remains uncertain and warrants further investigation. Third, several potentially relevant variables (e.g., sarcopenia, socioeconomic status, nutritional indices, and caregiver support) were not captured in our dataset. We also lacked detailed information regarding preoperative interventions, such as nutritional optimization or prehabilitation, which may have influenced postoperative outcomes. Fourth, because our complication data were extracted from a structured institutional registry that systematically records events requiring active intervention, minor Grade I complications might be underreported. However, this primarily affects non-actionable events and does not alter the assessment of clinically significant postoperative major outcomes. Fifth, this study did not directly compare the G8 with other established frailty assessment tools, limiting conclusions regarding its relative performance. Finally, a positive G8 result should not be interpreted as a definitive diagnosis of frailty. Rather, the G8 is a rapid screening tool designed to identify older patients who may be vulnerable and who may benefit from further comprehensive geriatric assessment. Accordingly, the present study should be interpreted as evaluating the prognostic value of a positive G8 screening result, rather than frailty as defined by a full geriatric assessment.

Future research should include prospective, multicenter validation of the G8 in UTUC, direct comparisons with alternative frailty measures, and interventional trials evaluating whether preoperative optimization strategies can mitigate frailty-associated risks and improve surgical and oncologic outcomes.

## Conclusions

The G8 screening tool provides a simple, objective, and practical approach to assess frailty in older patients with UTUC undergoing RNU. G8-low(score ≤ 14) identifies individuals at increased risk of postoperative complications and poor survival. Integrating G8-based frailty screening into preoperative evaluation can improve surgical risk prediction, inform treatment decisions—including consideration of nephron-sparing surgery—and support tailored perioperative care.

## Supplementary Information

Additional file 1.

## Data Availability

The data that support the findings of this study are available from National Cheng Kung University Hospital but restrictions apply to the availability of these data, which were used under license for the current study, and so are not publicly available. Data are however available from the authors upon reasonable request and with permission of National Cheng Kung University Hospital.

## Abbreviations

5-item FI: five—item frailty index
ASA: American Society of Anesthesiologists
BC: Bladder cancer
BMI: Body mass index
CGA: Comprehensive geriatric assessment
CI: Confidence interval
CSS: Cancer—specific survival
DM: Diabetes mellitus
eGFR: Estimated glomerular filtration rate
ESRD: End—stage renal disease
G8: Geriatric 8
GNRI: Geriatric Nutritional Risk Index
HR: Hazard ratio
HTN: Hypertension
IQR: Interquartile range
IRB: Institutional Review Board
LVI: Lymphovascular invasion
MIS: Minimally—invasive surgery
NSS: Nephron—sparing surgery
OR: Odds ratio
OS: Overall survival
PFS: Progression—free survival
pT: Pathological T stage
RNU: Radical nephroureterectomy
SD: Standard deviation
SPSS: Statistical Package for the Social Sciences
UC: Urothelial carcinoma
UTUC: Upper tract urothelial carcinoma
