# Supplementary material for: Geriatric 8 score predicts surgical risk and oncologic outcomes in older patients with upper tract urothelial carcinoma undergoing radical nephroureterectomy
Source: BMC Geriatr. 2026 Jul 7;26:1230. doi: 10.1186/s12877-026-07954-1 (PMC13625474; doi:10.1186/s12877-026-07954-1)
Supplement: Supplementary file 1 [file 12877_2026_7954_MOESM1_ESM.docx]

**Table S1. Description of Complications with Clavien-Dindo Grade 2 or Greater**

| **Complications No.** | **Non-frail (%) N=160** | **Frail (%) N=178** | **Total (%) N=338** |
| --- | --- | --- | --- |
| **Clavien-Dindo Grade II** | **57 (35.6%)** | **107 (60.1%)** | **164 (48.5%)** |
| Transfusion  Urinary tract infection  Delirium  Low urinary tract symptoms  Postoperative delayed bowel function/ Ileus  Acute kidney failure  Fever without focus  Bacteremia  Hyponatremia  Hyperkalemia  Cardiac arrhythmia  Postoperative hyperglycemia  Mild postoperative dyspnea  Surgical site infection  Parenteral nutrition  Wound poor healing  Cellulitis  Pneumonia  Metabolic acidosis  Postoperative hypotension requiring vasopressor  Extremities weakness and numbness  Gastritis | 35 (21.9%)  9 (5.6%)  2 (1.3%)  4 (2.5%)  2 (1.3%)  1 (0.6%)  1 (0.6%)  0 (0%)  0 (0%)  1 (0.6%)  0 (0%)  2 (1.3%)  1 (0.6%)  1 (0.6%)  0 (0%)  0 (0%)  0 (0%)  0 (0%)  0 (0%)  1 (0.6%)  0 (0%)  1 (0.6%) | 81 (45.5%)  11 (6.2%)  11 (6.2%)  5 (2.8%)  5 (2.8%)  4 (2.2%)  3 (1.7%)  3 (1.7%)  3 (1.7%)  2 (1.1%)  2 (1.1%)  0 (0%)  0 (0%)  0 (0%)  1 (0.6%)  1 (0.6%)  1 (0.6%)  1 (0.6%)  1 (0.6%)  0 (0%)  1 (0.6%)  0 (0%) | 116 (34.9%)  20 (5.9%)  13 (3.8%)  9 (2.7%)  7 (2.1%)  5 (1.5%)  4 (1.2%)  3 (0.9%)  3 (0.9%)  3 (0.9%)  2 (0.6%)  2 (0.6%)  1 (0.3%)  1 (0.3%)  1 (0.3%)  1 (0.3%)  1 (0.3%)  1 (0.3%)  1 (0.3%)  1 (0.3%)  1 (0.3%)  1 (0.3%) |
| **Clavien-Dindo Grade III** | **4 (2.5%)** | **16 (9.0%)** | **20 (5.9%)** |
| Major vessel bleeding  Surgical site dehiscence  Retroperitoneal abscess  **Vascular access thrombosis**  Blood clot evacuation  Upper GI bleeding, requiring endoscopic intervention  Postoperative delayed bleeding  Pneumothorax  In-hospital fall-related fracture | 1 (0.6%)  1 (0.6%)  1 (0.6%)  0 (0%)  1 (0.6%)  0 (0%)  0 (0%)  0 (0%)  0 (0%) | 3 (1.7%)  2 (1.1%)  2 (1.1%)  3 (1.7%)  1 (0.6%)  2 (1.1%)  1 (0.6%)  1 (0.6%)  1 (0.6%) | 4 (1.2%)  3 (0.9%)  3 (0.9%)  3 (0.9%)  2 (0.6%)  2 (0.6%)  1 (0.3%)  1 (0.3%)  1 (0.3%) |
| **Clavien-Dindo Grade IV** | **2 (1.3%)** | **12 (6.7%)** | **14 (4.2%)** |
| Shock  Unstable Hemodynamic requiring ICU care  Atrial Fibrillation with Rapid Ventricular Response  Metabolic acidosis requiring ICU care | 1 (0.6%)  1 (0.6%)  0 (0%)  0 (0%) | 8 (4.5%)  2 (1.1%)  1 (0.6%)  1 (0.6%) | 9 (2.7%)  3 (0.9%)  1 (0.3%)  1 (0.3%) |

**Table S2. Univariate and multivariate Cox regression analyses for predicting overall survival in patients with upper tract urothelial carcinoma after radical nephroureterectomy.**

| Variables | Univariate HR (95% CI) | p-value | Multivariate HR (95% CI) | p-value |
| --- | --- | --- | --- | --- |
| Age (≥80 vs <80) | 1.78 (1.23–2.59) | 0.002 | 2.07 (1.38–3.11) | <0.001 |
| Gender (Male vs Female) | 1.50 (1.04–2.15) | 0.03 | 1.45 (0.98–2.14) | 0.06 |
| Renal function (ml/min/1.73m2) |  |  |  |  |
| eGFR ≥ 45 | Reference |  | Reference |  |
| eGFR < 45 | 1.00 (0.66–1.52) | 1.00 |  |  |
| ESRD | 1.28 (0.79–2.08) | 0.31 |  |  |
| Previous/Concomitant BC (Yes vs No) | 1.44 (0.99–2.10) | 0.06 | 1.27 (0.84–1.91) | 0.26 |
| Tumor location |  |  |  |  |
| Renal pelvis | Reference |  | Reference |  |
| Ureter | 1.02 (0.67–1.54) | 0.94 | 1.67 (1.05–2.66) | 0.03 |
| Both | 1.60 (1.00–2.56) | 0.05 | 1.44 (0.87–2.37) | 0.15 |
| Pathological T stage |  |  |  |  |
| pTa/1 | Reference |  | Reference |  |
| pT2 | 2.21 (1.07–4.58) | 0.03 | 2.22 (1.04–4.77) | 0.04 |
| pT3/4 | 6.71 (3.81–11.82) | <0.001 | 5.19 (2.78–9.69) | <0.001 |
| Lymph node (N+ vs. Nx/0) | 4.44 (2.53–7.79) | <0.001 | 2.37 (1.18–4.73) | 0.015 |
| Tumor grade (High vs. Low) | 1.24 (0.46–3.36) | 0.67 |  |  |
| Lymphovascular invasion (Presence) | 3.84 (2.65–5.58) | <0.001 | 1.90 (1.21–2.99) | 0.006 |
| Adjuvant chemotherapy  (yes vs. no) | 2.39 (1.59-3.60) | <0.001 | 1.21 (0.77-1.90) | 0.416 |
| G8 (low vs. high) | 1.85 (1.27–2.70) | 0.001 | 1.77 (1.18–2.67) | 0.006 |

Abbreviations: HR = hazard ratio; CI = confidence interval; eGFR = estimated glomerular filtration rate; ESRD = end-stage renal disease; BC = bladder cancer.

**Table S3. Univariate and multivariate Cox regression analyses for predicting cancer-specific survival in patients with upper tract urothelial carcinoma after radical nephroureterectomy.**

| Variables | Univariate HR (95% CI) | p-value | Multivariate HR (95% CI) | p-value |
| --- | --- | --- | --- | --- |
| Age (≥80 vs <80) | 1.73 (1.18–2.53) | 0.005 | 1.92 (1.27–2.92) | 0.002 |
| Gender (Male vs. Female) | 1.51 (1.04–2.21) | 0.03 | 1.47 (0.99–2.19) | 0.06 |
| Renal function (ml/min/1.73m2) |  |  |  |  |
| eGFR ≥ 45 | Reference |  | Reference |  |
| eGFR < 45 | 0.99 (0.64–1.52) | 0.95 |  |  |
| ESRD | 1.24 (0.75–2.04) | 0.41 |  |  |
| Previous/Concomitant BC (Yes vs. No) | 1.45 (0.98–2.14) | 0.06 | 1.23 (0.81–1.88) | 0.34 |
| Tumor location |  |  |  |  |
| Renal pelvis | Reference |  | Reference |  |
| Ureter | 0.95 (0.62–1.46) | 0.81 | 1.55 (0.96–2.50) | 0.08 |
| Both | 1.60 (0.99–2.59) | 0.06 | 1.39 (0.83–2.31) | 0.21 |
| Pathological T stage |  |  |  |  |
| pTa/1 | Reference |  | Reference |  |
| pT2 | 2.45 (1.10–5.47) | 0.03 | 2.51 (1.09–5.78) | 0.03 |
| pT3/4 | 8.24 (4.39–15.45) | <0.001 | 5.97 (3.01–11.84) | <0.001 |
| Lymph node (N+ vs. Nx/0) | 4.68 (2.66–8.24) | <0.001 | 2.29 (1.15–4.59) | 0.02 |
| Tumor grade (High vs. Low) | 1.60 (0.51–5.04) | 0.42 |  |  |
| Lymphovascular invasion (Presence) | 4.17 (2.84–6.11) | <0.001 | 1.98 (1.25–3.14) | 0.004 |
| Adjuvant chemotherapy  (yes vs. no) | 2.51 (1.66-3.78) | <0.001 | 1.23 (0.78-1.93) | 0.382 |
| G8 (low vs. high) | 2.06 (1.39–3.07) | <0.001 | 2.02 (1.32–3.10) | 0.001 |

Abbreviations: HR = hazard ratio; CI = confidence interval; eGFR = estimated glomerular filtration rate; ESRD = end-stage renal disease; BC = bladder cancer.

**Table S4. Univariate and multivariate Cox regression analyses for predicting progression-free survival in patients with upper tract urothelial carcinoma after radical nephroureterectomy.**

| Variables | Univariate HR (95% CI) | p-value | Multivariate HR (95% CI) | p-value |
| --- | --- | --- | --- | --- |
| Age (≥80 vs <80) | 1.59 (1.14–2.23) | 0.007 | 1.74 (1.21–2.50) | 0.003 |
| Gender (Male vs Female) | 1.69 (1.21–2.36) | 0.002 | 1.57 (1.11–2.22) | 0.011 |
| Renal function (ml/min/1.73m2) |  |  |  |  |
| eGFR ≥ 45 | Reference |  | Reference |  |
| eGFR < 45 | 1.24 (0.85–1.80) | 0.27 |  |  |
| ESRD | 1.28 (0.82–2.00) | 0.28 |  |  |
| Previous/Concomitant BC (Yes vs No) | 1.34 (0.95–1.90) | 0.10 | 1.24 (0.86–1.80) | 0.25 |
| Tumor location |  |  |  |  |
| Renal pelvis | Reference |  | Reference |  |
| Ureter | 1.13 (0.77–1.66) | 0.53 | 1.79 (1.17–2.72) | 0.007 |
| Both | 1.80 (1.17–2.77) | 0.007 | 1.53 (0.98–2.38) | 0.06 |
| Pathological T stage |  |  |  |  |
| pTa/1 | Reference |  | Reference |  |
| pT2 | 2.08 (1.11–3.89) | 0.02 | 1.91 (0.99–3.67) | 0.053 |
| pT3/4 | 6.24 (3.85–10.10) | <0.001 | 4.08 (2.38–6.98) | <0.001 |
| Lymph node (N+ vs. Nx/0) | 5.78 (3.40–9.84) | <0.001 | 2.62 (1.40–4.91) | 0.003 |
| Tumor grade (High vs. Low) | 1.67 (0.62–4.50) | 0.32 |  |  |
| Lymphovascular invasion (Presence) | 4.09 (2.90–5.75) | <0.001 | 2.09 (1.38–3.17) | <0.001 |
| Adjuvant chemotherapy  (yes vs. no) | 2.98 (2.06-4.32) | <0.001 | 1.66 (1.12-2.46) | 0.012 |
| G8 (low vs. high) | 1.66 (1.18–2.33) | 0.004 | 1.70 (1.18–2.46) | 0.005 |

Abbreviations: HR = hazard ratio; CI = confidence interval; eGFR = estimated glomerular filtration rate; ESRD = end-stage renal disease; BC = bladder cancerr.
